# Tranexamic acid after aneurysmal subarachnoid hemorrhage is associated with worse outcome in males: post hoc analysis of the ULTRA trial

**DOI:** 10.1007/s00415-026-14037-z

**Published:** 2026-08-19

**Authors:** Raoul J. Koopmans, Maud A. Tjerkstra, Menno R. Germans, Mervyn D. I. Vergouwen, Korné Jellema, Radboud W. Koot, Nyika D. Kruyt, Jasper F. C. Wolfs, Hans Kieft, Dharmin Nanda, Bram van der Pol, Gerwin Roks, Frank de Beer, Loes J. A. Reichman, Paul J. A. M. Brouwers, Vincent I. H. Kwa, Taco C. van der Ree, Henri P. Bienfait, Hieronymus D. Boogaarts, Catharina J. M. Klijn, René van den Berg, Bert A. Coert, Janneke Horn, Charles B. L. M. Majoie, Gabriël J. E. Rinkel, Yvo B. W. E. M. Roos, W. Peter Vandertop, René Post, Dagmar Verbaan

**Affiliations:** 1 Department of Neurosurgery, Amsterdam UMC Location University of Amsterdam, Meibergdreef 9, PO Box 22660, 1100 DD Amsterdam, the Netherlands; 2 Amsterdam Neuroscience, Neurovascular Disorders, Amsterdam, the Netherlands; 3 Department of Neurosurgery, Clinical Neuroscience Center, University Hospital Zurich, Zurich, Switzerland; 4 Department of Neurology and Neurosurgery, UMC Utrecht Brain Centre, University Medical Centre Utrecht, Utrecht University, Utrecht, the Netherlands; 5 Department of Neurology, Haaglanden Medical Centre, The Hague, the Netherlands; 6 Department of Neurosurgery, Leids University Medical Centre, Leiden, the Netherlands; 7 Department of Neurology, Leids University Medical Centre, Leiden, the Netherlands; 8 Department of Neurosurgery, Haaglanden Medical Centre, The Hague, the Netherlands; 9 Department of Intensive Care, Isala Hospital, Zwolle, the Netherlands; 10 Department of Neurosurgery, Isala Hospital, Zwolle, the Netherlands; 11 Department of Neurosurgery, Elisabeth Tweesteden Ziekenhuis, Tilburg, the Netherlands; 12 Department of Neurology, Elisabeth Tweesteden Ziekenhuis, Tilburg, the Netherlands; 13 Department of Neurology, Spaarne Gasthuis, Haarlem, the Netherlands; 14 Department of Neurology, Ziekenhuisgroep Twente, Almelo, the Netherlands; 15 Department of Neurology, Medisch Spectrum Twente, Enschede, the Netherlands; 16 Department of Neurology, OLVG, Amsterdam, the Netherlands; 17 Department of Neurology, Dijklander Hospital, Hoorn, the Netherlands; 18 Department of Neurology, Gelre Hospital, Apeldoorn, The Netherlands; 19 Department of Neurosurgery, Radboud University Medical Centre, Nijmegen, the Netherlands; 20 Department of Neurology, Donders Institute for Brain, Cognition and Behaviour, Radboud University Medical Centre, Nijmegen, the Netherlands; 21 Department of Radiology and Nuclear Medicine, Amsterdam UMC, University of Amsterdam, Neuroscience Amsterdam, Meibergdreef 9, Amsterdam, Netherlands; 22 Department of Intensive Care, Amsterdam UMC, University of Amsterdam, Neuroscience Amsterdam, Meibergdreef 9, Amsterdam, Netherlands; 23 Department of Neurology, Amsterdam UMC, University of Amsterdam, Meibergdreef 9, Amsterdam, the Netherlands

**Keywords:** Subarachnoid hemorrhage, Hemocoagulation, Antifibrinolytic therapy, Tranexamic acid, Sex differences, Treatment outcome

## Abstract

**Background:**

The ULTRA trial demonstrated no improvement in clinical outcome at 6 months in patients with subarachnoid hemorrhage (SAH) when treated with ultra-early and short-term tranexamic acid (TXA). In this post hoc analysis, we evaluated the effect of TXA in females and males on clinical outcome after aneurysmal SAH (aSAH).

**Methods:**

The ULTRA trial was a prospective, randomized, controlled, open-label trial, conducted between 2013 and 2020. Patients with spontaneous SAH were assigned to ultra-early, short-term TXA in addition to usual care, or usual care only. In this study, we only included ULTRA participants with radiologically proven aSAH. We analyzed the potential effect of sex on the association between treatment group and clinical outcome by assessing effect modification, and evaluated the effect of TXA separately in females and males. The primary endpoint was clinical outcome at 6 months, assessed by the distribution of the modified Rankin Scale (mRS). Odds ratios (OR) and adjusted OR (aOR) with 95% confidence intervals (CI) were calculated using ordinal regression analyses. Secondary outcomes included good (mRS score 0–3) and excellent (mRS score 0–2) clinical outcome at 6 months, all-cause mortality at 30 days, all-cause mortality at 6 months, and in-hospital complications.

**Results:**

Of the 812 patients, 577 (71%) were female, of whom 286 (50%) received TXA. Of the 235 males, 109 (46%) received TXA. Significant effect modification of sex on the relation between treatment group and clinical outcome was observed (*p* = 0.044) in all aSAH patients. No significant differences in outcomes were observed among females. In males, ordinal regression analysis showed a difference in mRS distribution at 6 months to the detriment of males treated with TXA (aOR 1.94; 95% CI 1.20–3.14). No significant differences were observed between male treatment groups in secondary outcome measures.

**Discussion:**

In this post hoc analysis of the ULTRA trial, we observed a potential association between ultra-early, short-term TXA treatment and worse clinical outcomes in males at 6 months. Conversely, we did not observe a significant association between TXA treatment and clinical outcome in females. Consequently, these findings reinforce that routine use of TXA cannot be recommended in either females or males.

**Trial registration:**

The trial was registered on ClinicalTrials.gov (NCT02684812).

**Supplementary Information:**

The online version contains supplementary material available at https://doi.org/10.1007/s00415-026-14037-z.

## Introduction

Aneurysmal subarachnoid hemorrhage (aSAH) accounts for a high case fatality rate and survivors have high morbidity rates [1–3]. Rebleeding from a ruptured aneurysm is thought to be a result of dissolution of the blood clot by fibrinolysis at the site of aneurysm rupture [4], and is a major complication that increases the risk of poor outcome and mortality [3]. Therefore, it is recommended to minimize this risk by surgical or endovascular obliteration of the aneurysm as early as feasible [5–7]. Although early obliteration is endeavored, rebleeding rates remain high due to the substantial risk of rebleeding within the first 24 h, and the technical and logistic complexity of aneurysm obliteration therapy within this timespan [8].

In previous trials, antifibrinolytic therapy demonstrated a reduction in the occurrence of rebleeding, but no improvement in clinical outcome, possibly by an increase in the occurrence of delayed cerebral ischemia (DCI) due to long-term antifibrinolytic therapy, counteracting the beneficial effect of rebleed reduction [9, 10]. A recent systematic review on antifibrinolytic therapy after aSAH including 2717 participants [11] demonstrated a reduction in the occurrence of rebleeding (risk ratio (RR) 0.65, 95% confidence interval (CI) 0.47–0.91), without any improvement in clinical outcome or increased risk of developing DCI after the implementation of ischemia-preventative measures and < 72 h of treatment. In the ULTRA trial [12], ultra-early and short-term tranexamic acid (TXA) also showed no improvement in clinical outcome at 6 months. Nevertheless, possible beneficial or harmful effects of TXA within certain subgroups remain undisclosed.

Females are more susceptible to develop aSAH, although the exact etiology regarding the discrepancy in incidence between sexes remains unclear [13–15]. A meta-analysis showed that males had a higher risk of rebleeding than females [16], whereas a recent review demonstrated that delayed cerebral ischemia (DCI) was significantly more prevalent in females compared to males [17]. More dissimilarities in complications and outcome after aSAH between sexes are probable, but literature on these potential differences is scarce [16–21], and evidence leading to different treatment strategies absent.

In this post hoc analysis of the ULTRA trial, we investigated the effect of TXA on clinical outcome at 6 months after aSAH, in females and males separately.

## Methods

### Design

The protocol of the ULTRA trial has been published previously [12, 22, 23]. In summary, the ULTRA trial was a randomized, controlled, multicenter, open-label trial with blinded outcome assessment. Between July 2013 and January 2020, the trial was conducted in eight treatment centers and 16 referral hospitals in the Netherlands. The study was performed in accordance with the principles of the Declaration of Helsinki and International Conference of Harmonization guidelines for Good Clinical Practice and was registered on ClinicalTrials.gov (NCT02684812). The ethics committee of the Amsterdam University Medical Center (Amsterdam UMC, Amsterdam, the Netherlands) approved the trial protocol (2012_160#2012370).

### Patients and procedures

Adult patients admitted to one of the participating referring hospitals or treatment centers were included if they presented within 24 h of symptoms indicating subarachnoid hemorrhage (SAH), with non-contrast computed tomography (CT)-confirmed SAH. Exclusion criteria were no proficiency of the Dutch or English language; a perimesencephalic bleeding pattern on CT in combination with a Glasgow Coma Scale score of 13–15 without focal neurological deficit on admission or loss of consciousness; traumatic SAH pattern on CT; ongoing treatment for deep vein thrombosis (DVT) or pulmonary embolism (PE); a history of a hypercoagulability disorder; pregnancy; severe renal failure (serum creatinine > 150 µmol/l), or imminent death within 24 h. Directly after confirmation of SAH, patients were randomly assigned (in a 1:1 ratio) to either TXA treatment with usual care (TXA group), or usual care only (usual care group). Randomization was performed with a secured web-based system that stratified according to permuted blocks (random block sizes; maximum of 12) by treatment center. After randomization, patients in the TXA group immediately received a bolus of 1 g of TXA intravenously, followed by continuous intravenous infusion of 1 g every 8 h. Administration was continued for a maximum of 24 h, or until the start of aneurysm treatment. For the current post hoc analysis, we excluded patients with unknown received treatment as well as patients without a confirmed causative intracranial aneurysm on CT angiography (CTA) and/or digital subtraction angiography (DSA).

### Data collection

Baseline characteristics (i.e., sex, age, medication use (platelet inhibitors, anticoagulation, antihypertensive drugs), World Federation of Neurosurgical Societies (WFNS) grade [24] at admission, Fisher grade score [25] on first non-contrast head CT, aneurysm location, time from diagnosis to aneurysm treatment and treatment modality), common in-hospital complications following aSAH (i.e., suspected rebleeding after randomization and before aneurysm treatment, CT-confirmed rebleeding, DCI, hydrocephalus, per-procedural thrombo-embolic events, per-procedural aneurysm rupture, extracranial thrombosis, hemorrhagic complications, severe hyponatremia (< 125 mmol/L), pneumonia, bacterial meningitis, urinary tract infection, seizures, delirium, and Terson’s syndrome), mortality at 30 days, mortality at 6 months, and modified Rankin Scale (mRS) scores at 6 months were collected prospectively. A research nurse, trained according to a standard procedure and blinded to the randomization results, assessed the mRS scores by a standardized and validated telephone interview [26–28].

### Outcomes

For this post hoc analysis, the primary endpoint was clinical outcome at 6 months, assessed by the mRS score, ranging from 0 (no symptoms) to 6 (death). For the secondary outcomes, mRS scores were dichotomised into good (mRS 0–3) and poor (mRS 4–6) clinical outcome at 6 months, and into excellent (mRS 0–2) and non-excellent clinical outcome (mRS 3–6) at 6 months. Furthermore, secondary outcomes included all-cause mortality at 30 days and at 6 months, and the aforementioned in-hospital complications. The definitions of the complications are listed in the supplemental appendix of the ULTRA Trial [12].

### Statistical analysis

Included patients were analyzed in groups according to sex using the as-treated principle. We additionally performed intention-to-treat analyses. Normality of data was explored by a normal Q–Q plot and tested by the Shapiro–Wilk test (statistic test threshold 0.9). Baseline characteristics were assessed by sex in the overall study population and separately within female and male patients, and were summarized by descriptive statistics. Categorical variables are reported as percentages, normally distributed continuous variables as means with standard deviations, and non-normally distributed variables as medians with interquartile ranges (IQR). Group differences were analyzed using the independent *T*-test, Mann–Whitney U test, Chi-square test, or Fisher’s exact test, depending on the type and distribution of the data.

Effect modification of sex on the relation between TXA and clinical outcome was analyzed using ordinal regression analysis with clinical outcome as the dependent variable and the as-treated treatment group (TXA or usual care), sex, an interaction term (treatment group * sex), potential differences (*p* < 0.2) in baseline characteristics between females and males, and treatment center as independent variables. Effect modification was considered significant if the *p*-value of the interaction term was < 0.05.

For the primary outcome, ordinal regression analysis was used, and for secondary outcomes logistic regression analyses were used in females and males separately to calculate odds ratios (OR) and adjusted odds ratios (aOR), with a correction for the influence of treatment center and potential differences (*p* < 0.2) in baseline characteristics.

For the ordinal regression analysis, the proportional odds assumption was assessed using the test of parallel lines. In instances of formal violation, a sensitivity analysis was performed comparing the ordinal model to a multinomial logistic regression model. If the predicted probabilities and effect directions were comparable between models, the ordinal model was retained for parsimony and to facilitate clinical interpretation by providing a common odds ratio. If they were not comparable, the multinomial logistic regression model was presented. Furthermore, in case of violation of the proportional odds assumption, statistical significance was corroborated using the non-parametric Mann–Whitney *U* test (shift analysis), which does not rely on distributional assumptions.

Statistical analyses were performed using the IBM SPSS Statistics version 25 software (IBM Corporation, Armonk, NY, USA).

## Results

**Fig. 1:**
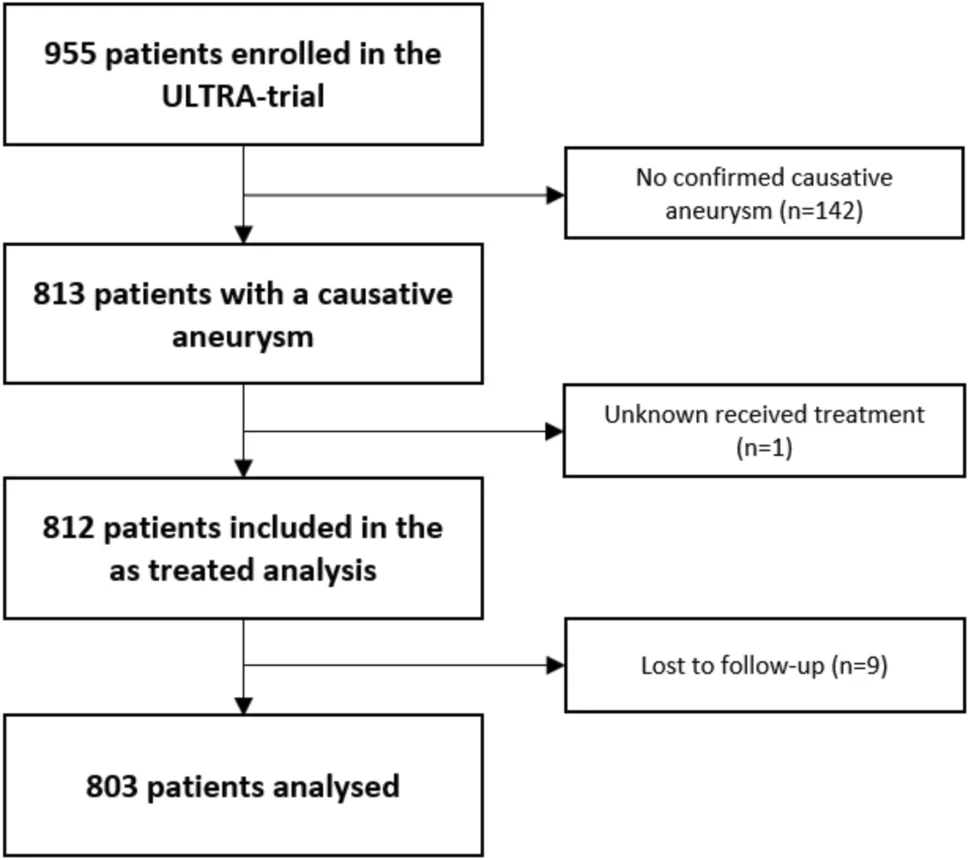
Flowchart of included patients

Of the 955 participants enrolled in the ULTRA trial, 813 patients had confirmed aSAH. Of these patients, 578 (71%) were female. The as-treated analyses included 812 patients, since one female was excluded due to unknown received treatment (Fig. 1). Primary outcome was obtained in 803 (99%) patients, because six females and three males were lost to follow-up. Baseline characteristics in females and males, with regard to treatment groups, showed no significant differences between groups (Table 1). In addition, baseline characteristics were compared between females and males irrespective of treatment group and are presented in the supplemental data (Table S1). In all aSAH patients, effect modification of sex on TXA was observed (*p* = 0.044) with respect to clinical outcome at 6 months by ordinal regression analysis after adjustment for treatment center, age, platelet inhibitor, and anticoagulation use.

**Table 1:** Baseline characteristics of 235 male and 577 female aSAH patients according to the treatment group (as-treated)

	Females *N* = 577			Males *N* = 235		
	TXA group *N* = 286	Usual care group *N* = 291	Significance *p*-value	TXA group *N* = 109	Usual care group *N* = 126	Significance *p*-value
Age (years), mean (SD)	58.8 (12.7)	59.6 (11.9)	0.40	57.5 (12.8)	55.6 (12.7)	0.25
Medication use^a^						
Platelet inhibitor	31 (11)	29 (10)	0.71	17 (16)	20 (16)	0.95
Anticoagulation	10 (4)	6 (2)	0.29	3 (3)	8 (6)	0.19
Antihypertensive drugs	68 (24)	69 (24)	0.95	26 (24)	25 (20)	0.46
None of the above	195 (69)	203 (70)	0.78	74 (68)	85 (67)	0.94
WFNS^b^			0.81			0.33
I	87 (31)	102 (35)		39 (36)	46 (37)	
II	58 (21)	52 (18)		19 (18)	23 (18)	
III	14 (5)	13 (5)		9 (8)	3 (2)	
IV	63 (22)	65 (23)		21 (20)	30 (24)	
V	59 (21)	56 (19)		19 (18)	24 (19)	
Fisher grade score			0.62			0.18
II	16 (6)	12 (4)		5 (5)	3 (2)	
III	72 (25)	80 (27)		27 (25)	44 (35)	
IV	198 (69)	199 (68)		77 (71)	79 (63)	
Aneurysm location			0.99			0.47
Anterior circulation	233 (82)	238 (82)		85 (78)	103 (82)	
Posterior circulation	52 (18)	53 (18)		24 (22)	23 (18)	
Time to treatment (h), median (IQR)	14 (6–20)	15 (5–21)	0.70	13 (6–20)	12 (4–20)	0.47
Treatment modality ^c^			0.42			0.88
Endovascular	196 (69)	185 (64)		68 (63)	80 (63)	
Clipping	57 (20)	64 (22)		24 (22)	30 (24)	
None	33 (12)	42 (14)		16 (15)	16 (13)	

Data presented as *n* (%), unless noted otherwise. Percentages may not total 100 because of rounding

^a^Medication use: missing in four females

^b^WFNS score could not be assessed in eight females (1.4%) and two males (0.9%)

^c^One patient with two potentially causative aneurysms, of which one was clipped and one was treated endovascularly

*aSAH* Aneurysmal subarachnoid hemorrhage, *TXA* tranexamic acid, *SD* standard deviation, *WFNS* World Federation of Neurosurgical Societies, *IQR* interquartile range

### Females

Of 577 females, 286 (50%) were treated with TXA. The mRS scores at 6 months were obtained in 571 of 577 (99%) females. TXA showed no significant effect on overall distribution of mRS scores at 6 months (OR 1.01, 95% CI 0.75–1.35). After adjustment for treatment center, the aOR remained 1.01 (95% CI 0.75–1.35), confirmed by the Mann–Whitney *U* test (*p* = 0.967). In addition, no significant differences between groups were observed regarding secondary endpoints (Table 2) or in-hospital complications (Table 3). The intention-to-treat analyses demonstrated comparable results (supplemental data, Table S2).

**Table 2:** Primary outcome (modified Rankin Scale score at 6 months) and secondary outcomes in 577 females after aSAH according to treatment group (as-treated)

	TXA group (*N* = 286)	Usual care group (*N* = 291)	OR (95% CI)^†^	aOR (95% CI)
Primary outcome				
mRS scores at 6 months*	NA	NA	1.01 (0.75–1.35)	1.01 (0.75–1.35)
mRS 0	8 (3)	11 (4)	NA	NA
mRS 1	24(8)	23 (8)	NA	NA
mRS 2	102 (36)	112 (39)	NA	NA
mRS 3	37 (13)	24 (8)	NA	NA
mRS 4	19 (7)	6 (2)	NA	NA
mRS 5	21 (7)	33 (11)	NA	NA
mRS 6	72 (25)	79 (27)	NA	NA
Secondary outcomes				
Good clinical outcome* (mRS 0–3)	171 (60)	170 (59)	1.06 (0.76–1.48)	1.06 (0.76–1.48)
Excellent clinical outcome* (mRS 0–2)	134 (47)	146 (51)	0.88 (0.63–1.22)	0.88 (0.63–1.22)
All-cause mortality at 30 days	61 (21)	70 (24)	0.86 (0.58–1.27)	0.85 (0.58–1.26)
All-cause mortality at 6 months	72 (25)	79 (27)	0.90 (0.62–1.31)	0.90 (0.62–1.31)

Data presented as *n* (%), unless noted otherwise. Percentages may not total 100 because of rounding

*Six females lost to follow-up; tranexamic acid group (*N* = 283); usual care group (*N* = 288)

^†^The proportional odds assumption was formally violated in the female subgroup. However, sensitivity analysis using a multinomial model demonstrated that the ordinal model provides a valid summary estimate of the treatment effect. The distributional shift was further confirmed by the non-parametric Mann–Whitney *U* test (*p* = 0. 0.967)

*aSAH* Aneurysmal subarachnoid hemorrhage, *TXA* tranexamic acid, *OR* odds ratio, *aOR* adjusted odds ratio, with a correction for the influence of treatment centers, *95*% *CI* 95% confidence interval

**Table 3:** Complications during hospital admissions in 577 females after aSAH according to the treatment group (as-treated)

	TXA group (*N* = 286)	Usual care group (*N* = 291)	OR (95% CI)
Any complication	237 (83)	251 (86)	0.77 (0.49–1.21)
All^a^ rebleedings before aneurysm treatment	30 (10)	43 (15)	0.68 (0.41–1.11)
CT-proven rebleedings	24 (8)	38 (13)	0.61 (0.36–1.05)
Hydrocephalus	187 (65)	179 (62)	1.18 (0.84–1.66)
Delayed cerebral ischemia^b^	73 (26)	82 (28)	0.87 (0.60–1.26)
Thrombo-embolic complications during endovascular treatment	19 (10)	27 (15)	0.63 (0.34–1.17)
Infarct related to clipping	15 (26)	13 (20)	1.40 (0.60–3.27)
Aneurysm rupture during procedure	24 (9)	25 (10)	0.94 (0.52–1.69)
- Coiling	13 (7)	11 (6)	1.12 (0.49–2.58)
- Clipping	11 (19)	14 (22)	0.85 (0.35–2.07)
Extra-cranial thrombosis	3 (1)	5 (2)	0.61 (0.14–2.56)
- Deep venous thrombosis	0 (0)	0 (0)	–
- Pulmonary embolism	2 (1)	5 (2)	0.40 (0.08–2.09)
Hemorrhagic complication^c^	17 (6)	22 (8)	0.78 (0.40–1.49)
Severe hyponatremia (< 125 mmol/L)	10 (3)	6 (2)	1.72 (0.62–4.80)
Pneumonia	35 (12)	42 (14)	0.83 (0.51–1.34)
Bacterial meningitis	21 (7)	21 (7)	1.02 (0.54–1.91)
Urinary tract infection	29 (10)	34 (12)	0.85 (0.51–1.44)
Seizures	39 (14)	32 (11)	1.28 (0.78–2.11)
Delirium	34 (12)	38 (13)	0.90 (0.55–1.47)
Terson	12 (4)	13 (4)	0.94 (0.42–2.09)
Other complication	82 (29)	91 (31)	0.88 (0.62–1.26)

Data presented as *n* (%), unless noted otherwise

^a^Both suspected (not CT proven) and CT proven

^b^Data missing in one patient; usual care group (*N* = 290)

^c^Data missing in one patient; TXA group (*N* = 285)

*TXA* Tranexamic acid, *OR* odds ratio, *95*% *CI* 95% confidence interval, *aSAH* aneurysmal subarachnoid hemorrhage

### Males

**Fig. 2:**
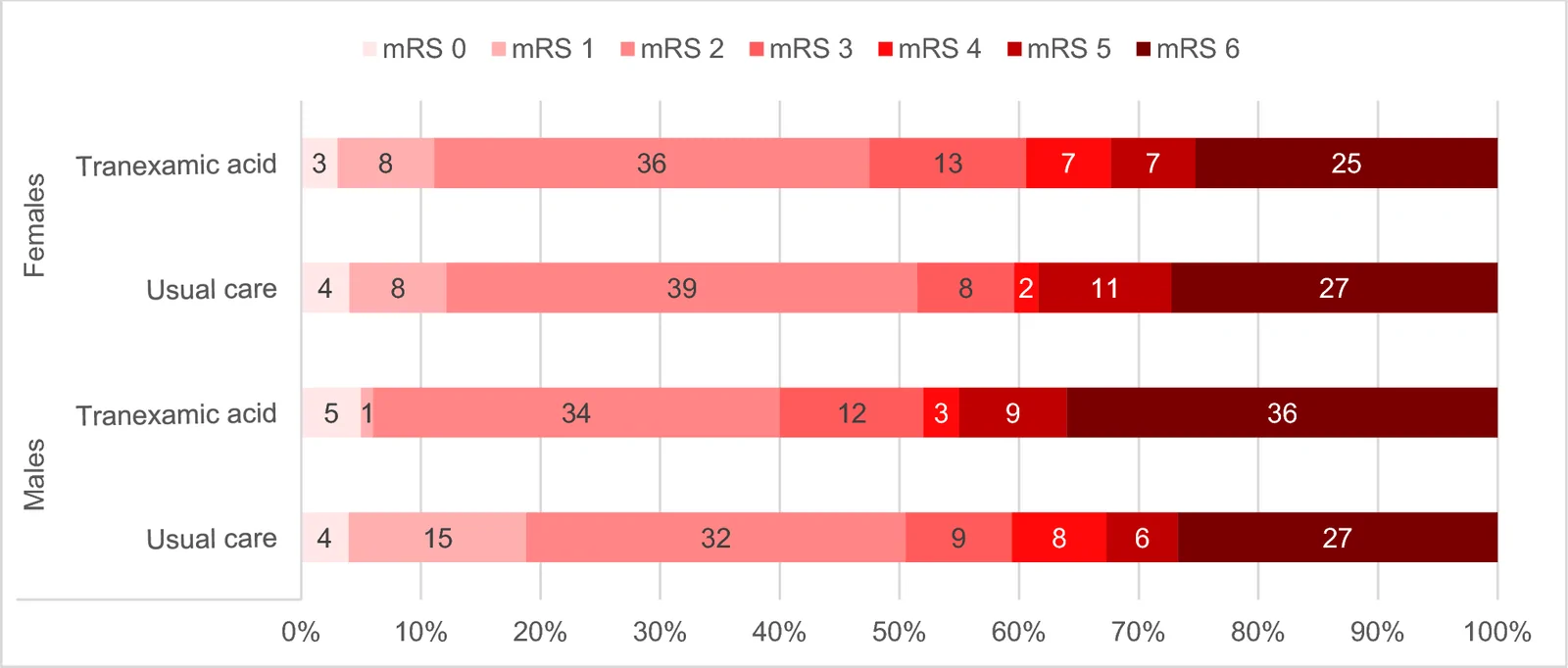
Primary outcome (distribution of modified Rankin Scale score at six months) in 577 females and 235 males with aSAH according to treatment group (as-treated). Stacked bar chart of modified Rankin Scale (mRS) scores (0–6). 0 no symptoms; 1 no clinically significant disability; 2 slight disability; 3 moderate disability; 4 moderately severe disability; 5 severe disability; 6 death. Aneurysmal subarachnoid hemorrhage (aSAH), odds ratio (OR), 95% confidence interval (95% CI) Data presented as %. Percentages may not total 100 because of rounding. Six females lost to follow up; Tranexamic acid group (*N* = 283); Usual care group (*N* = 288). Three males lost to follow-up; Tranexamic acid group (*N* = 108); Usual care group (*N* = 124). In females, the overall distribution of mRS scores six months after aSAH demonstrated no significant differences between treatment groups (OR 1.01, 95% CI 0.75–1.35; aOR 1.01, 95% CI 0.75–1.35), confirmed by the Mann–Whitney U test (*p* = 0.967). In males, the overall distribution of mRS scores six months after aSAH demonstrated significant worse mRS scores in the TXA group, compared to the usual care group (OR 1.72, 95% CI 1.08–2.74; aOR 1.94, 95% CI 1.20–3.14), confirmed by the Mann–Whitney *U* test (*p* = 0.023)

Of 235 males, 109 (46%) were treated with TXA. The mRS scores at 6 months were obtained in 233 of 235 (99%) males. The overall distribution of mRS scores at 6 months showed worse clinical outcome in males treated with TXA compared to males not treated with TXA (OR 1.72, 95% CI 1.08–2.74). After adjustment for treatment center, Fisher grade score on first non-contrast head CT and anticoagulation use (*p* < 0.2), the aOR was 1.94 (95% CI 1.20–3.14), confirmed by the Mann–Whitney *U* test (*p* = 0.023) (Table 4; Fig. 2). There were no significant differences regarding secondary endpoints or in-hospital complications between groups, although non-significant lower rates of good and excellent clinical outcome at 6 months, non-significant higher rates of mortality at 30 days and at 6 months, in-hospital complications, hydrocephalus, DCI, per-procedural thrombo-embolic events, seizures, delirium, and Terson’s syndrome were observed in males treated with TXA (Table 5). The intention-to-treat analyses demonstrated comparable results (supplemental data, Table S3).

**Table 4:** Primary outcome (modified Rankin Scale score at 6 months) and secondary outcomes in 235 males after aSAH according to the treatment group (as-treated)

	TXA group (*N* = 109)	Usual care group (*N* = 126)	OR (95% CI)^†^	aOR (95% CI)
Primary outcome				
mRS scores at 6 months*	NA	NA	1.72 (1.08–2.74)	1.94 (1.20–3.14)
mRS 0	5 (5)	5 (4)	NA	NA
mRS 1	1 (1)	18 (15)	NA	NA
mRS 2	37 (34)	40 (32)	NA	NA
mRS 3	13 (12)	11 (9)	NA	NA
mRS 4	3 (3)	10 (8)	NA	NA
mRS 5	10 (9)	7 (6)	NA	NA
mRS 6	39 (36)	33 (26)	NA	NA
Secondary outcomes				
Good clinical outcome* (mRS 0–3)	56 (52)	74 (60)	0.73 (0.43—1.23)	0.71 (0.39—1.29)
Excellent clinical outcome* (mRS 0–2)	43 (40)	63 (51)	0.64 (0.38—1.08)	0.61 (0.34—1.10)
All-cause mortality at 30 days	32 (29)	29 (23)	1.39 (0.78—2.50)	1.33 (0.71—2.47)
All-cause mortality at 6 months	39 (36)	33 (26)	1.57 (0.90—2.74)	1.59 (0.87—2.87)

Data presented as *n* (%), unless noted otherwise. Percentages may not total 100 because of rounding

*Three males lost to follow-up; tranexamic acid group (*N* = 108); usual care group (*N* = 124)

^†^The proportional odds assumption was formally violated in the male subgroup. However, sensitivity analysis using a multinomial model demonstrated that the ordinal model provides a valid summary estimate of the treatment effect. The distributional shift was further confirmed by the non-parametric Mann–Whitney *U* test (*p* = 0.023)

*aSAH* Aneurysmal subarachnoid hemorrhage, *TXA* tranexamic acid, *OR* odds ratio, *aOR* adjusted odds ratio, with a correction for the influence of treatment centers, Fisher grade score on first non-contrast head CT and anticoagulation use, *95*% *CI* 95% confidence interval

**Table 5:** Complications during hospital admissions in 235 males after aSAH according to treatment group (as-treated)

	TXA group (*N* = 109)	Usual care group (*N* = 126)	OR (95% CI)
Any complication	95 (87)	103 (82)	1.52 (0.74—3.12)
All^a^ rebleedings before aneurysm treatment	16 (15)	22 (17)	0.81 (0.40—1.64)
CT-proven rebleedings	14 (13)	19 (15)	0.83 (0.40—1.75)
Hydrocephalus	64 (59)	68 (54)	1.21 (0.72—2.04)
Delayed cerebral ischemia	26 (24)	26 (21)	1.21 (0.65—2.23)
Thrombo-embolic complications during endovascular treatment	10 (15)	6 (8)	2.13 (0.73—6.19)
Infarct related to clipping	7 (29)	5 (17)	1.98 (0.54—7.29)
Aneurysm rupture during the procedure	7 (8)	11 (10)	0.74 (0.28—2.00)
- Coiling	2 (3)	2 (3)	1.18 (0.16—8.62)
- Clipping	5 (21)	9 (30)	0.61 (0.18—2.16)
Extra-cranial thrombosis	2 (2)	2 (2)	1.16 (0.16—8.37)
- Deep venous thrombosis	0 (0)	2 (2)	–
- Pulmonary embolism	2 (2)	0 (0)	–
Hemorrhagic complication	9 (8)	12 (10)	0.86 (0.35—2.11)
Severe hyponatremia (< 125 mmol/L)	1 (1)	2 (2)	0.57 (0.05—6.42)
Pneumonia	16 (15)	26 (21)	0.66 (0.33—1.31)
Bacterial meningitis	6 (6)	11 (9)	0.61 (0.22—1.71)
Urinary tract infection	7 (6)	8 (6)	1.01 (0.36—2.89)
Seizures	13 (12)	9 (7)	1.76 (0.72—4.29)
Delirium	20 (18)	18 (14)	1.35 (0.67—2.70)
Terson	6 (6)	4 (3)	1.78 (0.49—6.47)
Other complication	33 (30)	35 (28)	1.13 (0.64—1.99)

Data presented as *n* (%), unless noted otherwise

^a^Both suspected (not CT proven) and CT proven

*aSAH* Aneurysmal subarachnoid hemorrhage, *TXA* tranexamic acid, *OR* odds ratio, *95*% *CI* 95% confidence interval

## Discussion

In this post hoc analysis of the ULTRA trial, ultra-early and short-term TXA treatment was associated with worse clinical outcome at 6 months in males. In females, treatment with TXA did not improve, or worsen, clinical outcome at 6 months.

In the ULTRA trial [12, 29], ultra-early and short-term TXA showed no improvement in clinical outcome at 6 months within the (a)SAH population as a whole. However, possible beneficial or detrimental effects of TXA within certain subgroups remained undisclosed. One might hypothesize that TXA could have a negative effect on clinical outcome in females due to a possible increase in DCI rate [17], without the potency of improving clinical outcome on lowering rebleeding rates, since these occur less frequently in females [16]. Conversely, in males who seem less prone to DCI [17], TXA might have a larger potential to lower rebleeding rates, whilst possible increasing DCI rates hold off. Thus, TXA leads to a hypothetical beneficial effect in males and a detrimental effect in females. These hypothetical effects of TXA (beneficial in males and detrimental in females) are not confirmed by our study.

In traumatic brain injury (TBI), TXA administration has extensively been investigated. The CRASH-3 trial [30], including 12,737 patients, demonstrated a reduction in head injury-related death in TBI patients when TXA was administered within 3 h after injury, without an increase in adverse events or complications. In patients suffering from mild-to-moderate TBI, the risk ratio was 0.78 (95% CI 0.64–0.95), while in patients with severe TBI, the reduction in risk was non-significant. A recent sex-disaggregated analysis on this large placebo-controlled trial [30, 31] demonstrated that TXA significantly reduced the relative risk (RR) of death within 24 h in males (RR 0.72, 95% CI 0.55–0.94) suffering from TBI, without any major extracranial bleeding. In females the reduction in RR was non-significant (RR 0.81, 95% CI 0.46–1.42).

The results of our study, however, are not in line with this sex-disaggregated analysis in TBI patients. Although no statistically significant differences in complications were observed to account for our findings, a sum of factors might do. The rates of DCI, hydrocephalus, seizures, and thrombo-embolic complications during endovascular treatment and related to clipping were higher in males treated with TXA, but separately did not reach statistical significance. A recent systematic review on antifibrinolytic therapy [11], including both studies on short-term and long-term administration of TXA, demonstrated an increase in DCI within the aSAH population. However, this effect was reduced and demonstrated no significant association after implementation of ischemia-preventative measures and short-term administration of antifibrinolytic therapy (RR 1.10; *P* = 0.49), which is consistent with our results in males. Additionally, this systematic review demonstrated a non-significant increased rate of reported hydrocephalus (RR 1.09, 95% CI 0.99–1.20), while another meta-analysis on the safety and efficacy of TXA in SAH (32) showed a significant increase in hydrocephalus within the overall pooled results in the TXA group (RR 1.13, 95% CI 1.02–1.24). This might suggest a possible negative effect of TXA in male clinical outcome as a result of a reduction in plasminogen activity, leading to diminished absorption of intraventricular hemorrhage and cerebral spinal fluid, causing hydrocephalus [32, 33].

With regard to TXA and seizures, it is known that moderate to high dose of intravenous TXA is associated with seizures, due to binding of TXA on GABA receptors, abolishing GABA-mediated inhibition [34]. Studies in cardiac surgery demonstrated an increase in postoperative generalized seizures with an OR of 14.3 (95% CI 5.5–36.7; *p* < 0.001) and a 2.5 times higher in-hospital mortality rate [35–37]. Although the TXA dosage used in cardiac surgery exceeded ours (1–4 g per day), and TXA-associated seizures are dose dependent [38], significant increased rates have been described in patients without brain injury receiving more than 2 g per day [39]. In addition, another recent post hoc subgroup analysis of the ULTRA trial [40] demonstrated an increased seizure rate in the TXA group compared to the usual care group (17% versus 10% (OR 1.96, 95% CI 1.03–3.73)) in patients suffering from poor-grade aSAH. This might suggest that brain injury lowers the threshold for TXA-associated seizures.

In summary, it is conceivable that TXA may negatively affect clinical outcome in males through a collective effect of increased rates of hydrocephalus, DCI, other thrombo-embolic complications, and seizures. However, given the absence of statistically significant differences in these outcomes, this interpretation remains speculative. Future studies are warranted to formally investigate these potential mechanisms.

The strengths of this study are the large sample size and the participation of 24 hospitals throughout the Netherlands, attenuating patient-, clinician and hospital-specific outcomes, leading to more generalizable results. In addition, outcome assessment was standardized and blinded, and few patients were lost to follow-up. This study has some limitations. In this post hoc analysis, we subdivided the aSAH population according to sex, resulting in smaller sample sizes and consequently lower power, also for detecting interaction effects, which increases the possibility of chance findings. Furthermore, by only using a selection of the ULTRA participants, randomization was undone, which could have led to differences between treatment groups, although the baseline characteristics were evenly distributed. Moreover, the Netherlands is a small and densely populated country, in which the average distance to treatment centers is low. This might result in relatively early admission and obliteration therapy in the Netherlands, and a subsequent lower risk of rebleeding which could detract from the generalizability of our results.

## Conclusion

In this post hoc analysis of the ULTRA trial, we observed a potential association between ultra-early, short-term TXA treatment and worse clinical outcomes in males at 6 months. Conversely, we did not observe a significant association between TXA treatment and clinical outcome in females. Consequently, these findings show that routine use of TXA cannot be recommended in either females or males.

## Supplementary Information

Below is the link to the electronic supplementary material.

Supplementary Material 1

## Data Availability

All data and material requests should be submitted to RJK for consideration. Access to anonymized data may be granted following review.

## Declarations

### Reporting checklist

The authors have used the CONSORT checklist as a reporting checklist for this article.
