# Supplementary material for: Tranexamic acid after aneurysmal subarachnoid hemorrhage is associated with worse outcome in males: post hoc analysis of the ULTRA trial
Source: J Neurol. 2026 Aug 19;273(9):528. doi: 10.1007/s00415-026-14037-z (PMC13490208; doi:10.1007/s00415-026-14037-z)
Supplement: Supplementary file 1 [file 415_2026_14037_MOESM1_ESM.docx]

**Supplementary Appendix**

**Table of content**

**Results**

Table S1. Baseline characteristics of 235 male and 577 female aSAH patients. Page 2

Table S2. Intention-to-treat analysis regarding primary outcome (modified Rankin Scale score at six months) and secondary outcomes in 578 females after aSAH according to treatment group. Page 3

Table S3. Intention-to-treat analysis regarding primary outcome (modified Rankin Scale score at six months) and secondary outcomes in 235 males after aSAH according to treatment group. Page 4

**Table S1.** Baseline characteristics of 235 male and 577 female aSAH patients.

|  | Females  N=577 | Males  N=235 | Significance p-value |
| --- | --- | --- | --- |
| Age (years), *mean (SD)* | 59.2 (12.3) | 56.5 (12.7) | 0.005 |
| Medication use ^a^ |  |  |  |
| Platelet inhibitor | 60 (10) | 37 (16) | 0.04 |
| Anticoagulation | 16 (3) | 11 (5) | 0.18 |
| Antihypertensive drugs | 137 (24) | 51 (22) | 0.50 |
| None of the above | 398 (69) | 159 (68) | 0.62 |
| WFNS ^b^ |  |  | 0.91 |
| I | 189 (33) | 85 (36) |  |
| II | 110 (19) | 42 (18) |  |
| III | 27 (5) | 12 (5) |  |
| IV | 128 (22) | 51 (22) |  |
| V | 115 (20) | 43 (18) |  |
| Fisher Grade Score |  |  | 0.40 |
| II | 28 (5) | 8 (3) |  |
| III | 152 (26) | 71 (30) |  |
| IV | 397 (69) | 156 (66) |  |
| Aneurysm location |  |  | 0.56 |
| Anterior circulation | 471 (82) | 188 (80) |  |
| Posterior circulation | 105 (18) | 47 (20) |  |
| Time to treatment (hours), *median (IQR)* | 14 (6-21) | 12 (5-20) | 0.44 |
| Treatment modality ^c^ |  |  | 0.74 |
| Endovascular | 381 (66) | 148 (63) |  |
| Clipping | 121 (21) | 54 (23) |  |
| None | 75 (13) | 32 (14) |  |

Data presented as n (%), unless noted otherwise. Percentages may not total 100 because of rounding. Aneurysmal subarachnoid hemorrhage (aSAH), Tranexamic acid (TXA), Standard deviation (SD), World Federation of Neurosurgical Societies (WFNS), interquartile range (IQR). ^a^  Medication use: missing in four females. ^b^ WFNS score could not be assessed in eight females (1.4%) and two males (0.9%). ^c^ One patient with two potentially causative aneurysms, of which one was clipped and one was treated endovascularly.

**Table S2.** Intention-to-treat analysis regarding primary outcome (modified Rankin Scale score at six months) and secondary outcomes in 578 females after aSAH according to treatment group.

|  | **TXA group**  (N=297) | **Usual care group**  (N=281) | **OR (95% CI)** | **aOR (95% CI)** |
| --- | --- | --- | --- | --- |
| **Primary outcome** | | | | |
| **mRS scores at six months*** | NA | NA | 1.09 (0.81 - 1.47) | 1.09 (0.81 - 1.47) |
| **mRS 0** | 8 (3) | 11 (4) | NA | NA |
| **mRS 1** | 24 (8) | 23 (8) | NA | NA |
| **mRS 2** | 105 (35) | 109 (39) | NA | NA |
| **mRS 3** | 37 (12) | 24 (9) | NA | NA |
| **mRS 4** | 19 (6) | 6 (2) | NA | NA |
| **mRS 5** | 23 (8) | 31 (11) | NA | NA |
| **mRS 6** | 78 (26) | 74 (26) | NA | NA |
| **Secondary outcomes** | | | | |
| **Good clinical outcome**^*^ **(mRS 0-3)** | 174 (59) | 167 (60) | 0.96 (0.69 - 1.35) | 0.96 (0.69 - 1.35) |
| **Excellent clinical outcome**^*^ **(mRS 0-2)** | 137 (47) | 143 (51) | 0.82 (0.59 - 1.14) | 0.82 (0.59 - 1.14) |
| **All-cause mortality at 30 days** | 63 (21) | 69 (25) | 0.83 (0.56 - 1.22) | 0.83 (0.56 - 1.22) |
| **All-cause mortality at six months** | 78 (26) | 74 (26) | 1.00 (0.69 - 1.44) | 1.00 (0.69 - 1.44) |

Data presented as n (%), unless noted otherwise. Percentages may not total 100 because of rounding. Aneurysmal subarachnoid hemorrhage (aSAH), Tranexamic acid (TXA), odds ratio (OR), adjusted odds ratio (aOR) with a correction for the influence of treatment centers, 95% confidence interval (95% CI). *Six females lost to follow-up; Tranexamic acid group (N=294); Usual care group (N=278).

**Table S3.** Intention-to-treat analysis regarding primary outcome (modified Rankin Scale score at six months) and secondary outcomes in 235 males after aSAH according to treatment group.

|  | **TXA group**  (N=112) | **Usual care group**  (N=123) | **OR (95% CI)** | **aOR (95% CI)** |
| --- | --- | --- | --- | --- |
| **Primary outcome** | | | | |
| **mRS scores at six months*** | NA | NA | **1.63 (1.02 - 2.59)** | **1.92 (1.19 - 3.10)** |
| **mRS 0** | 5 (4) | 5 (4) | NA | NA |
| **mRS 1** | 1 (1) | 18 (15) | NA | NA |
| **mRS 2** | 39 (35) | 38 (31) | NA | NA |
| **mRS 3** | 14 (13) | 10 (8) | NA | NA |
| **mRS 4** | 4 (4) | 9 (7) | NA | NA |
| **mRS 5** | 9 (8) | 8 (7) | NA | NA |
| **mRS 6** | 39 (35) | 33 (27) | NA | NA |
| **Secondary outcomes** | | | | |
| **Good clinical outcome**^*^ **(mRS 0-3)** | 59 (53) | 71 (59) | 0.80 (0.48 - 1.34) | 0.76 (0.42 - 1.39) |
| **Excellent clinical outcome* (mRS 0-2)** | 45 (41) | 61 (50) | 0.67 (0.40 - 1.13) | 0.61 (0.34 - 1.11) |
| **All-cause mortality at 30 days** | 32 (29) | 29 (24) | 1.30 (0.72 - 2.33) | 1.26 (0.68 - 2.35) |
| **All-cause mortality at six months** | 39 (35) | 33 (27) | 1.46 (0.84 - 2.54) | 1.50 (0.83 - 2.71) |

Data presented as n (%), unless noted otherwise. Percentages may not total 100 because of rounding. Aneurysmal subarachnoid hemorrhage (aSAH), Tranexamic acid (TXA), odds ratio (OR), adjusted odds ratio (aOR) with a correction for the influence of treatment centers, Fisher grade score on first non-contrast head CT and anticoagulation use. 95% confidence interval (95% CI). *Three males lost to follow-up; Tranexamic acid group (N=111); Usual care group (N=121).
